# Supplementary material for: Derivation of Human Extraembryonic Mesoderm-like Cells from Primitive Endoderm
Source: Int J Mol Sci. 2023 Jul 12;24(14):11366. doi: 10.3390/ijms241411366 (PMC10380231; doi:10.3390/ijms241411366)
Supplement: Supplementary file 1 [file ijms-24-11366-s001.zip › Supplementary Table S1.pdf]

Supplementary Table S1: List of primers

Gene	Forward	Reverse
ACTB	CCAACCGCGAGAAGATGA	CCAGAGGCGTACAGGGATAG
GAPDH	AGCCACATCGCTCAGACAC	GCCCAATACGACCAAATCC
ISL1	GCAGCCCAATGACAAAACTAAT	CCGTCGTGTCTCTCTGGACT
LIX1	GGCAGACCCAAGATGGACAGA	CCACCACACCTTCACTTGGGA
LUM	ATCCCTGGTTGAGCTGGATCTG	TTGCCATCCAAACGCAAATGCT
OCT4	GCAGCAGATCAGCCACATCG	AAAGGAGACCCAGCAGCCTC
TBXT	GCTGTGACAGGTACCCAACC	CATGCAGGTGAGTTGTCAGAA
TFAP2B	CAGCACACAGACCCGAGTGA	CGGCTGTTCCCTATCGGTGT
VGLL1	GACACGGCAGCAAGACATCC	GATTGGGGAGGCAAGTGGGT
